# Centrality Analysis of Protein-Protein Interaction Networks and Molecular Docking Prioritize Potential Drug-Targets in Type 1 Diabetes

**DOI:** 10.22037/ijpr.2020.113342.14242

**Published:** 2020

**Authors:** Asma Soofi, Mohammad Taghizadeh, Seyyed Mohammad Tabatabaei, Mostafa Rezaei Tavirani, Heeva Shakib, Saeed Namaki, Nahid Safari Alighiarloo

**Affiliations:** a *Department of Physical Chemistry, School of Chemistry, College of Sciences, University of Tehran, Tehran, Iran. *; b *Bioinformatics Department, Institute of Biochemistry and Biophysics, Tehran University, Tehran, Iran. *; c *Medical Informatics Department, School of Medicine, Mashhad University of Medical Sciences, Mashhad, Iran. *; d *Proteomics Research Center, Department of Basic Science, Faculty of Paramedical Sciences, Shahid Beheshti University of Medical Sciences, Tehran, Iran. *; e *Cellular and Molecular Endocrine Research Center, Research Institute for Endocrine Sciences, Shahid Beheshti University of Medical Sciences, Tehran, Iran.*; f *Immunology Department, Faculty of Medical Sciences, Shahid Beheshti University of Medical Sciences, Tehran, Iran. *; g *Endocrine Research Center, Institute of Endocrinology and Metabolism, Iran University of Medical Sciences, Tehran, Iran.*

**Keywords:** Type 1 diabetes, Systems biology approach, Protein-protein interaction network, Topological centrality, Molecular docking

## Abstract

Type 1 diabetes (T1D) occurs as a consequence of an autoimmune attack against pancreatic β- cells. Due to a lack of a clear understanding of the T1D pathogenesis, the identification of effective therapies for T1D is the active area in the research. The study purpose was to prioritize potential drugs and targets in T1D via systems biology approach. Gene expression data of peripheral blood mononuclear cells (PBMCs) and pancreatic β-cells in T1D were analyzed and differential expressed genes were integrated with protein-protein interactions (PPI) data. Multiple topological centrality parameters of extracted query-query PPI (QQPPI) networks were calculated and the interaction of more central proteins with drugs was investigated. Molecular docking was performed to further predict the interactions between drugs and the binding sites of targets. Central proteins were identified by the analysis of PBMC (MYC, ERBB2, PSMA1, ABL1 and HSP90AA1) and pancreatic β-cells (HSP90AB1, ESR1, RELA, RAC1, NFKB1, NFKB2, IKBKE, ARRB2 and SRC) QQPPI networks. Thirteen drugs which targeted eight central proteins were identified by further analysis of drug-target interactions. Some drugs which investigated for diabetes treatment in the experimental models of T1D were prioritized by literature verification, including melatonin, resveratrol, lapatinib, geldanamycin, eugenol and fostaminib. Finally, according on molecular docking analysis, lapatinib-ERBB2 and eugenol-ESR1 exhibited highest and lowest binding energy, respectively. This study presented promising results for the prioritization of potential drug-targets which might facilitate T1D targeted therapy and its drug discovery process more effectively.

## Introduction

Type 1 diabetes (T1D) is characterized by partial or absolute insulin deficiency as a result of chronic immune-mediated destruction of pancreatic β-cells, leading to hyperglycemia and consequent polyuria, polydipsia, and weight loss (1). At first glance, it seems like a single autoimmune disease but it presumably results from a complex interplay between environmental factors and microbiome, genome, metabolism, and immune systems with individual variations (2). The mainstay treatment for T1D is daily injections or continuous subcutaneous infusion of insulin to control blood sugar (3). Despite advances in the production, purification, formulation, and insulin delivery methods, it is difficult for patients to achieve optimal glucose control. Therefore, various adjunctive therapies for patients with T1D are available (4). Further, the other important aspect of T1D management is pancreatic β-cells preservation. In this line, numerous clinical trials have uncovered how immune modulation can impede the β-cell loss either by blocking the autoimmune response or by re-establishing immune tolerance (3, 5). Despite noticeable improvement in patients’ survival and health, especially in the past 25 years, a treatment for T1D is elusive (6). 

Nowadays, scientific researches show noticeable shift to systems-based understanding of molecular mechanism underlying biological resources, which remarkably affected drug discovery studies and represented the necessity of movement from traditional pharmacology (7). Systems pharmacology applies systems biology principles and combines high throughput experimental studies with computational analysis to study drugs, drug targets, and drug effects (8). Network-based gene expression profiling constructed by integrating multiple factors including disease genes, gene expression intensities and proteins network provides efficient strategy to discover therapeutic signatures. 

Transcriptome analysis of the target organ, *i.e*., pancreatic β-cells, and peripheral blood mononuclear cells (PBMCs) are informative entities for the investigation of genes expression profiles in T1D (9, 10). Till date, gene expression profiles have been integrated with protein-protein interactions (PPI) data to ameliorate their predictive performance (11-13). Networks of interacting proteins in diseases’ pathways have been analyzed for identifying candidate drug targets (14-16). Since well-connected proteins in the PPI networks have more potential to play crucial role in a cellular function, their targeting would be more effective rather than ordinary proteins.

The main goal of a network analysis is the connecting of a network structure to a function. For instance, different classes of disease genes presented distinction features based on their connectivity patterns in the human PPI network analysis (17, 18). The topological analysis of PPI networks provides a whole view of the network and helps to identification of the components with a central role in the network connectivity (19-21). Investigating of the network centrality measures can be a source of essential nodes’ discovery in various species’ interactomes (22). In this line, Peng *et al*. study explored standard pathways and gene expression-based pathways’ information and showed how network centrality measures have been effective in reorganization of potential therapeutic targets. They also alter the network topology, which resulted in different therapeutic targets discovery and introduced tissue-specific data (23). It has been also reported that topologically closer genes in PPI networks, *i.e*., having a lower shortest path distance in a network, were more regulated by structurally similar drugs rather than dissimilar drugs (24). 

Here, we integrated gene expression profiles with interactome (protein-protein interactions at the whole genome level) data to construct PPI networks using abnormally expressed genes in paired pancreatic β-cells and PBMCs in T1D. Topologically central proteins in PPI networks were identified. Candidate drugs and targets were then prioritized from the analysis of central proteins and drugs interactions. Docking analysis disclosed the interactions between candidate drugs and targets at the atomic level.

## Exprimental

*Gene expression data processing*

Gene expression profiles of PBMCs (GSE9006) and pancreatic β-cells (GSE35296) were used to determine abnormally expressed genes in T1D (19). A total of 43 newly diagnosed T1D patients and 24 healthy subjects were present in GSE9006 dataset. Gene expression profile of pancreatic β-cells was provided from human isolated islet samples which cultured under normal conditions as well as in the presence of cytokines (IL-1B and IFN-γ). According to our earlier work, GSE9006 data (HG-U133A and HG-U133B) was normalized by RMA algorithm in the affy package within R software and differentially expressed genes were determined by ANOVA test (*p* < 0.05). For each gene, the minimal *p*-value (between HG-U133A and HG-U133B) was chosen. In the case of GSE35296, up and downregulated genes were extracted by Fisher’s exact test and p-values were corrected by the Benjamin–Hochberg procedure (*p* < 0.05) (9, 10 and 19).

*PPI network construction and centrality* *analysis*

Experimentally proved PPI data provided by International Molecular Exchange (IMEx) consortium were obtained from IntAct MINT DIP databases (25-27). Differentially expressed genes in PBMCs and pancreatic β-cells were separately located on the human PPI network to construct Query-Query PPI (QQPPI) networks which involved the direct interactions among the query proteins as the nodes. The QQPPI networks were visualized with the yFiles organic layout algorithm in Cytoscape software (28). The following five more important centrality parameters were calculated using CentiBiN software to determine biologically significant nodes (29). Degree centrality is the number of links to a given node. In PPI networks, nodes with a higher degree are considered as hubs and they are usually located at the center of the network (30). Betweenness centrality measures the number of shortest paths passing through a node within a network. High betweenness nodes, named as bottlenecks, monitor the flow of information within a network (31). Closeness centrality calculates the average distance of all the shortest paths between a node and every other node within a network. High closeness should indicate the proximity of a node to all other nodes (31). Eigen vector centrality measures the relative significance of all nodes in the network. Thereby, a node that is connected to highly important nodes achieves more weight than a node that is connected to low important nodes. Since a node with a high eigenvector connects to numerous central nodes, it is considered the central and influential node (31). Centroid value is the most complex node centrality index and considers couples of nodes (*i*, *j*). The centroid value of a node *i* is the number of nodes with minimum shortest path which are closer to *i* than *j*. The highest centroid node has the highest number of neighbors separated by the shortest path to it (31). Centrality values of nodes were calculated and nodes were arranged in ascending order of the centrality values. Thirty nodes which had the highest values for each centrality parameter were identified. More central nodes had at least two high centrality parameters.

*Drug enrichment analysis*

Central nodes in both PBMCs and pancreatic β-cells QQPPI networks were screened as targets. To find potential drugs, central proteins were curated through a manual search in DrugBank database (version 5.4.1). The parameters were set as: FDA approved, experimental and investigational. This information was subsequently used to evaluate the association of drug-targets with T1D treatment by literature survey. 

*Molecular docking analysis*

Molecular docking has been widely used to predict the interactions between a small molecule and the binding site of the target at the atomic level. Docking analysis was performed for potential targets and their candidate drugs which investigated for diabetes treatment in experimental models (32-34). The crystallographic structures of reference targets (ESR1, ERBB2, RAC1 and HSP90AB1) were extracted from RCSB PDB (http://www.pdb.org/). The selected PDB IDs have following quality parameters. The ESR1 structure with PDB ID = 3ERT (resolution = 1.90 Å, R-value and R-free equal to 0.229 and 0.262, respectively) and the ERBB2 structure with PDB ID = 3PP0 (resolution = 2.25 Å, R-value and R-free equal to 0.185 and 0.260, respectively), the RAC1 structure with PDB ID = 3TH5 (resolution = 2.30 Å, R-value and R-free equal to 0.229 and 0.264, respectively), and the HSP90AB1 structure with PDB ID = 1UYM (resolution = 2.45 Å, R-value and R-free equal to 0.235 and 0.294, respectively), which warranted adequate quality for further docking analysis. 

**Figure 1:**
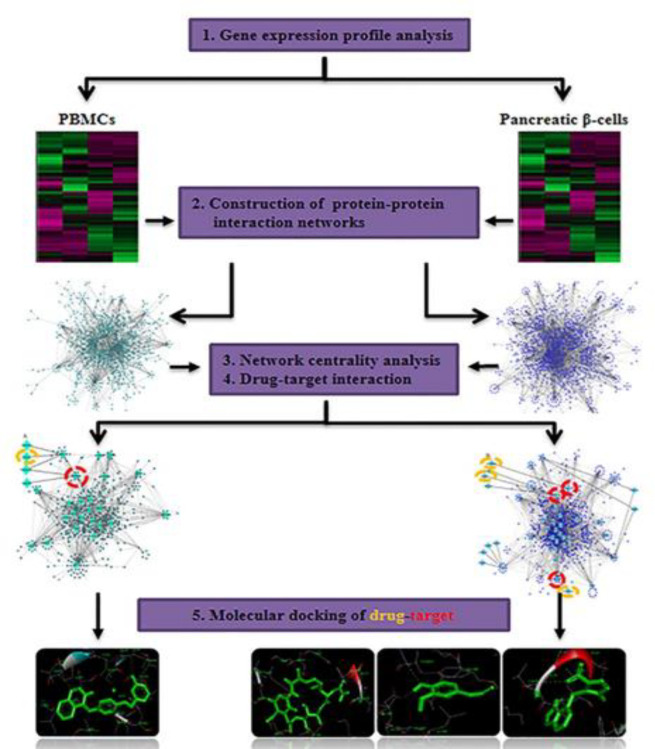
A workflow shows a network-based approach to prioritize drug-targets in T1D

The macromolecule structures were separated from the co-crystallized ligands and unnecessary water molecules were removed using Discovery Studio Visualizer 4.5. The structures of Lapatinib, Azathioprine, Geldanamycin, and Eugenol (PubChem CID 208908, 2265, 5288382, and 3314 respectively) were obtained from PubChem (https://pubch em.ncbi.nlm.nih.gov/). The 3D structures of the compounds were created by Openbabel 2.4.1 and different formats for both receptor and ligand molecules were converted using this software. Prior to any calculation, all 3D structures of ligands were initially energy minimized with MMFF94S force field using conjugate-gradient algorithm with 5000 run repetition. AutoDock-4 software in PyRx Virtual Screening tool was used to carry out molecular docking calculations. The protein coordinates were kept rigid, while the ligands were flexible and moved on the grid map which was set around the co-crystallographic ligands. In order to prepare the receptor molecules, polar hydrogens and kollman charges were added in AutoDock tool. The ligand was prepared by adding Gasteiger charges to each ligand atom. AutoDock 1.5.6.40 was utilized for the molecular docking simulation. Lamarckian genetic algorithm (LGA) with 150 independent runs per ligand was used to get best docking conformations. The number of individual population was set to 150. The max number of energy evaluations was set to 2,500,000 and the max number of generation was set to 27000. The quality of the docking has been assessed by removing co-crystallized ligands from the active site of each protein and re-docking into the binding pocket. A root mean square deviation (RMSD) was obtained from re-docking of each protein indicating that the docking procedure used in the current study could be relied upon to be capable of reproducing a similar conformation at the active site of each protein. Discovery Studio 4.5 Client was used for the visualization of docking results shows the study workflow. Figure 1 shows the study workflow.

## Results

*Identification of differential expressed genes*

Gene expression analysis was performed on PBMCs and pancreatic β-cells samples. In the case of PBMCs, 2466 genes were determined as differentially expressed ones (*p* < 0.05), of which 1024 were upregulated and 1442 were downregulated (Supplementary file 1). For pancreatic β-cells, 3068 genes were significantly differential expressed (*FDR* < 0.05), of these genes, 1416 were upregulated and 1652 were downregulated (9).

*PPI network construction and topological centrality analysis*

**Figure 2:**
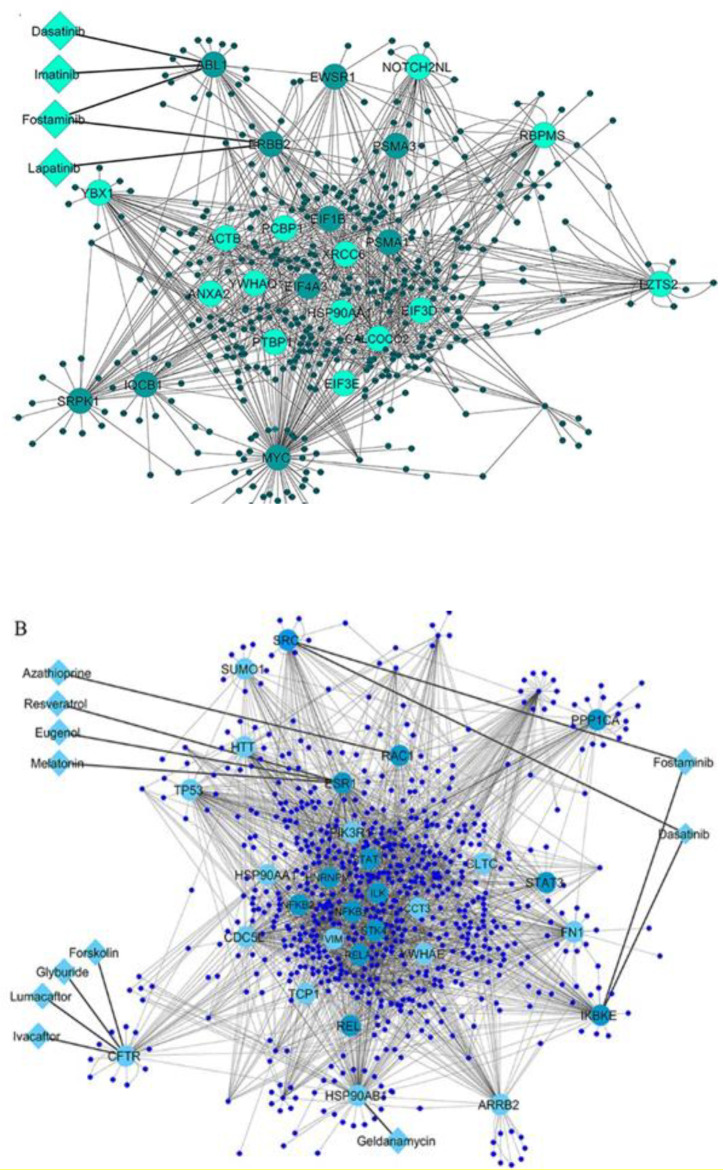
(A) PBMCs QQPPI network and (B) pancreatic β-cells QQPPI network. Protein-protein interaction (PPI) networks of differentially expressed genes which involved the first neighbors of central nodes. Nodes with high centrality measures are illustrated by bigger size and different colors than others. The interaction of drugs with targets is shown in each QQPPI network. Up-regulated and down-regulated genes are colored by light green and dark green, respectively in (A), and light blue and dark blue in (B)

To investigate the interactions among the differential expressed genes in T1D at the protein level, two QQPPI networks were established using the three IMEx databases. The QQPPI networks which included 949 proteins and 1776 interactions in PBMCs and 1358 proteins and 3505 interactions in pancreatic β-cells were used for further analysis. Five centrality parameters were measured for all the nodes presented in the QQPPI networks. MYC, YBX1, SRPK1, ERBB2, PSMA1, ABL1, HSP90AA1 and XRCC6 were more central nodes in PBMC QQPPI network. HSP90AB1, ESR1, CDC5L, RELA, RAC1, NFKB1, NFKB2, IKBKE, ARRB2, TP53, SRC, CFTR, HSP90AA1, PIK3R1 and PPP1CA were more central nodes in pancreatic β-cells QQPPI network. The list of nodes’ centrality values in PBMCs and pancreatic β-cells QQPPI networks were provided in Supplementary file 2 and 3, respectively. More central nodes of PBMCs and β-cells QQPPI networks are illustrated in (Figures 2A and Figure 2B), respectively. Besides, centrality parameters of fourteen more central proteins which identified as drug targets are provided in (Table 1).

*Prioritization of potential drugs and targets*

Twenty-three and thirty proteins in the high-confidence PBMCs and pancreatic β-cells QQPPI networks were curated for identifying of related drugs, respectively, using DrugBank database. At first screening, twenty-one drugs which targeted fourteen proteins obtained by this approach. Potential drugs and their protein targets are presented in Table 2; they were then verified if their associations with diabetes treatment were reported by the retrieved literatures (Table 2). The results showed that one drug, glyburide, was approved for the treatment of type 2 diabetes (T2D) and three drugs are being explored in several clinical trials for T1D (imatinib) and pre-diabetes or T2D (resveratrol and melatonin) treatment. In this line, the association of resveratrol and melatonin were studied in the experimental models of T1D. Azathioprine was earlier investigated by some clinical trials for T1D treatment. Both imatinib and azathioprine were immunosuppressive drugs. Lumacaftor/Ivafactor is being studied for glycemic control in cystic fibrosis related diabetes by clinical trials. Further, the association of six drugs with hyperglycemic or other diabetic condition has been reported in the experimental models of T1D or T2D, including, lapatinib, geldanamycin, eugenol, fostaminib, dasatinib and forskolin. Finally, four drug-target interactions (lapatinib-ERBB2, geldanamycin-HSP90AB1, azathioprine-RAC1 and eugenol-ESR1) were selected for further investigation by docking analysis. 

*Molecular docking and binding energy analysis*

Single molecular docking was carried out to explore the characteristics of the binding conformation and the interacting residues of four targets and candidate drugs related to diabetes. Autodock-4 was used in this study to repeatedly dock each ligand to the binding pocket of the target. The docking analysis resulted in 150 conformations for all 4 complexes, and the docked conformation corresponding to the lowest binding energy for each receptor-ligand complex was selected as the most probable binding conformation. The ligands were embedded within the active site of proper target protein individually. The formation of hydrogen bonds was observed in order to analyze the establishment of the active site of the target protein. The analysis of binding site residues lying within 4 Å distance of the ligand for each complex indicated that the drugs were surrounded by both hydrophobic and hydrophilic residues (Table 3). The binding pocket of all targets was generally surrounded by both hydrophobic and hydrophilic residues.

 Before docking the drug to a receptor structure, the docking protocol was validated by docking of the co-crystallized ligand into the binding pocket to obtain the docked pose. The RMSD in the range of 2–3 Å indicated appropriate docking, and as shown in the Supplementary file 4, overall docking conformations produced by Autodock-4 were within 0.5-1.5 Å of RMSD; this was showed the enough quality of the parameters for docking simulation in reproducing of the X-ray crystal structures. 

**Figure 3:**
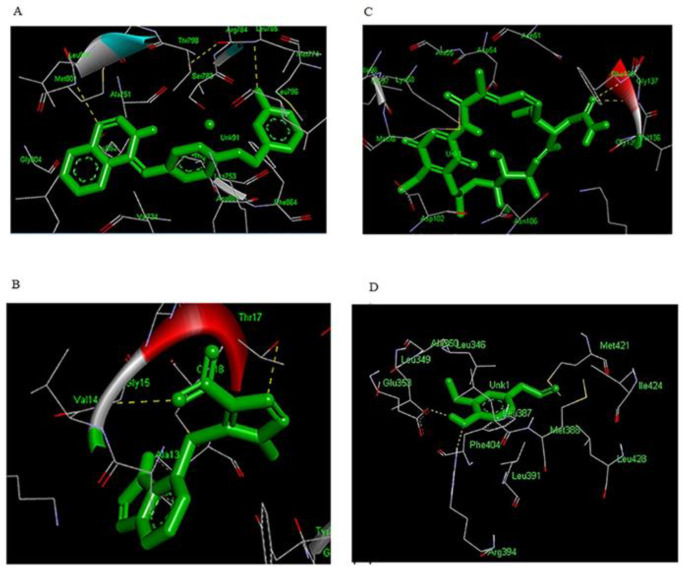
(A) Lapatinib-ERBB2 (B) Azathioprine-RAC1 (C) Geldanamycin-HSP90AB1 (D) Eugenol-ESR1. Molecular docking complexes. Drugs are shown in green stick model. H-bonds formed between residues and drugs are shown as yellow lines

Hydrogen bonds play a role in stabilizing the protein-ligand complex. The best conformation of each inhibitor at properly biological target formed great number of interactions with the main residues in the active site of the target. As shown in (Table 4), lapatinib was oriented inside the active site of ERBB2 in a way that formed hydrogen bond with residues LEU-785 (3.160Å) and MET-801 (3.149Å) of the loop region. Furthermore, the analysis of docked complex of lapatinib- ERBB2 showed binding energy of -10.59 kcal/mol (RMSD 1.16 Å), (Figure.3A). Lapatinib-ERBB2 complex showed highest binding energy among four complexes in this study. The analysis of docking complex of azathioprine-RAC1 revealed the binding energy of -9.32 kcal/mol (RMSD 2.0 Å), respectively and exhibited two binding interactions with GLY-15 (2.991 Å) and THR-17 (3.172 Å) residue of the loop region, (Table 4 and Figure 3B). The docked complex of geldanamycin-HSP90AB1showed binding affinities of -8.83 kcal/mol (RMSD 1.93 Å) which is bound with alpha helix residues such as GLY-137 (3.105 Å) and PHE-138 (3.06537 Å), (Table 4 and Figure 3C). In case of eugenol-ESR1, the complex has binding energy of -5.23 kcal/mol (RMSD 1.21 Å) and formed tow hydrogen bond with GLU-353 (1.791 Å) and ARG-394 (2.885 Å), (Table 4 and Figure.3D). Eugenol-ESR1 complex showed the lowest binding energy in compare to other complexes. 

## Discussion

Systems biology approach gives us a comprehensive view to improve our understanding of disease mechanisms and introduce new way for a discovery of novel drugs and repurposing of existing drugs (35). In this study, differentially expressed genes of PBMCs and pancreatic β-cells in T1D integrated with PPI data. Five centrality parameters, degree, betweenness, closeness, centroid value and eigenvector were measured for nodes in PBMCs and pancreatic β-cells QQPPI networks to find central proteins. A protein is considered a key molecule only if it is selected in at least two centrality measures. By analysis of central proteins and drugs interactions, several candidate drugs and targets were prioritized for T1D. Furthermore, some predicted drug-targets with a relevant to diabetes were used as an input for docking analysis which can calculate the probability of a physical interaction with the given drug and candidate targets. 

In the current study, fourteen targets and twenty-one candidate drugs were identified. By literature review, thirteen drugs which targeted eight key proteins showed association with diabetes. Some of them such glyburide has been approved for T2D and some ones like imatinib, resveratrol and melatonin are being evaluated in the clinical trials in patients with diabetes. Glyburide targets CFTR gene and it is second-generation sulfonylureas which approved for diabetes management. Glyburide is an antagonist of CFTR. The results of a study revealed a role of CFTR in glucose-induced electrical activities and insulin secretion in β-cells (36).  Grishman *et al*. study proposed a glyburide as one of the potential therapies to decrease the progression of T1D due its ability to decrease IL1β levels (37). Imatinib targets ABL gene. Endoplasmic reticulum stress in β-cell was increased in the NOD mouse as a result of the c-Abl tyrosine kinase activity. Consequently, the unfolded protein response was promoted that ultimately leading to β-cell death; this process might be affected via inhibition of c-Abl by imatinib (38). Moreover, insulin response was ameliorated in the experimental model of T1D by imatinib. Now, phase II clinical trial of imatinib treatment is ongoing in early-onset patients with T1D (39). Resveratrol targets ESR1 gene and is being evaluated in the clinical trials in patients with insulin resistance and T2D. Resveratrol binds to estrogen receptors which might be linked to the anti-diabetic effect in diabetes (40). It is well established that resveratrol diminish blood glucose levels in animals with experimental T1D (41). In this line, the results of a study demonstrated that resveratrol or 17β-estradiol apparently protected against STZ-induced diabetes in OVX mice; they probably improve antioxidant activities and islet function, promote muscle glucose uptake and prohibit the expression of p-ERK (42). Melatonin also targets ESR1 gene; it is being assessed in the clinical trials in patients with T2D. Melatonin interferes with estrogen-signaling pathways (43). The increased level of melatonin synthesis was reported in an animal model of STZ-induced T1D (44). The improvement of immune response and the anti-inflammatory effect were mentioned as the consequence of melatonin, which might inhibit the disease onset or ameliorate the survival of islet grafts transplanted for T1D therapy (45). 

 Moreover, four targets and their candidate drugs that their associations have been proved in the experimental models of diabetes were also prioritized. First, geldanamycin targets HSP90AB1 gene. Geldanamycin is specific HSP90 inhibitor. HSP90 inhibitors such as geldanamycin and its derivatives target HSP90 N terminus and block its ATPase activity; they have been identified as potential treatment strategy in cancer and promising drugs for immune and inflammatory diseases, including diabetes (46). The result of a study presented that heat shock proteins as well as treatment with geldanamycin noticeably improve diabetic macrophages activation, resulting in compromising mounting of inflammatory and immune responses (47). Besides, hyperglycemia was reversed by chronic dosing of HSP90 inhibitors in the diabetic db/db mouse model, and insulin sensitivity was made better in the diet-induced obese mouse model of insulin resistance (32). The second, eugnol targets ESR1. Eugenol is an estrogen receptor antagonist. Several medicinal applications were reported for the eugenol treatment such as antibacterial, antiviral, antioxidant, anti-inflamatory agent (48). The result of Al-Trad *et al*. study showed significant anti-oxidative and anti-inflammatory effect of eugenol in HFD/STZ-induced diabetic rats. Moreover, insulin sensitivity was improved by eugenol and skeletal muscle glucose uptake was stimulated via activation of the GLUT4-AMPK signaling pathway (33). The third, lapatinib targets ErbB2 gene. Lapatinib is a member of tyrosin kinase inhibitors (TKIs) which can target tyrosine kinase enzymes and interfere with downstream intracellular messaging pathways (49). Numerous studies represented the glucose-lowering potential of TKIs, which suggest careful attention to apply these drugs to patients with diabetes (50). Lapatinib is a dual inhibitor of EGFR and ErbB2 receptor tyrosine kinases, by which high glucose-induced apoptosis and vascular dysfunction were refined via resistance to signaling changes influenced by diabetes in the experimental T1D models (34, 51). The last, azathioprine targets Rac1 gene. Azathioprine suppresses both *T* and *B-lymphocyte* function (52). Tiede *et al.* reported that azathioprine induces immunosuppression by prohibition of Rac1 activation in T cells, which may clarify the beneficial immunosuppressive effects of azathioprine. Therefore, it might help to design the novel specific therapies for organ transplantation and autoimmune diseases (53). Furthermore, Veluthakal *et al*. identified a known inhibitor of Rac1, NSC23766, which remarkably suppresses reactive oxygen species (ROS) generation in pancreatic islet β-cells in *in-vitro*, and significantly inhibits the development of spontaneous diabetes in the NOD mice (54). Different trials were earlier conducted using azathioprine as an immunosuppressive drug to treat children newly diagnosed with T1D (52, 55). Although there were partly successful in improving metabolic outcomes in diabetic patients, the reported side effects made it unpopular. However, recently Geliebter *et al*. showed the first recent case reports exhibiting the possible positive effect of azathioprine in tertiary prevention of T1D (56). 

**Table 1:** Centrality parameters of targets extracted form PBMCs and pancreatic β-cells QQPPI networks

**Name**	**Degree**	**Betweenness** **Centrality**	**Closeness** **Centrality**	**Centroid value**	**Eigen vector**
**PBMCs**					
ABL1	27	0.0627	0.2917	-378	0.0605
ERBB2	32	0.0762	0.2942	-333	0.0758
MYC	117	0.3630	0.3584	319	0.6041
**Pancreatic β-cells**					
FN1	86	0.0985	0.3439	-203	0.1258
CFTR	35	0.0289	0.3007	-793	0.0499
IKBKE	69	0.0498	0.3276	-439	0.1760
NFKB1	35	0.0197	0.3329	-376	0.1219
NFKB2	55	0.0291	0.3495	-192	0.2166
PIK3R1	55	0.0292	0.3275	-356	0.0873
RAC1	36	0.0337	0.3162	-435	0.0362
RELA	74	0.0620	0.3525	-160	0.2143
SRC	46	0.0309	0.3195	-456	0.0772
HSP90AB1	112	0.1418	0.3686	64	0.2870
ESR1	98	0.1150	0.3600	-84	0.2335

**Table 2:** The list of potential drugs and their targets extracted from PBMCs and pancreatic β-cells QQPPI networks

**Gene name**	**DrugBank ID**	**Drug name**		**Actions**	**Drug group**
**PBMCs**					
ABL1	DB00619	Imatinib	inhibitor		Approved
	DB01254	Dasatinib	multitarget		approved, investigational
	DB04868	Nilotinib	inhibitor		approved, investigational
	DB12010	Fostamatinib	inhibitor		approved, investigational
ERBB2	DB01259	Lapatinib	inhibitor		approved, investigational
	DB12010	Fostamatinib	inhibitor		approved, investigational
MYC	DB08813	Nadroparin	inhibitor		approved, investigational
**Pancreatic β-cells**					
FN1	DB08888	Ocriplasmin	cleavage		Approved
CFTR	DB01016	Glyburide	antagonist		Approved
	DB00887	Bumetanide	antagonist		Approved
	DB08820	Ivacaftor	potentiator		Approved
	DB09280	Lumacaftor	modulator		Approved
	DB02587	Colforsin- Forskolin	inhibitor		experimental, investigational
IKBKE	DB12010	Fostamatinib	inhibitor		approved, investigational
NFKB1	DB08814	Triflusal	antagonist		approved, investigational
NFKB2	DB01296	Glucosamine	antagonist		approved, investigational
PIK3R1	DB01064	Isoprenaline	agonist		approved, investigational
RAC1	DB00993	Azathioprine	-		Approved
RELA	DB08908	Dimethyl fumarate	-		approved, investigational
SRC	DB01254	Dasatinib	multitarget		approved, investigational
	DB12010	Fostamatinib	inhibitor		approved, investigational
HSP90AB1	DB02424	Geldanamycin	-		experimental, investigational
ESR1	DB09086	Eugenol	-		Approved
	DB02709	Resveratrol	-		approved, experimental, investigational
	DB01065	Melatonin	antagonist		Approved

**Table 3:** The list of residues presented around the 4 Å distances of each ligand in a specific receptor after docking

**Drug**	**Receptor**	**Residues**
Lapatinib	ERBB2	LEU-726,VAL-734,TYR-735,ALA751,ILE752,LYS-753,ILE-767,GLU-770,ALA-771,MET-774,SER-783,ARG-784,LEU-796,THR-798-PRO-802,CYC-805,ASP-863,PHE-864
Azathioprine	RAC1	GLY-10,ASP-11,GLY-12,ALA-13,VAL-14,GLY-15,LYS-16,THR-17,CYS-18,LEU-19,PHE-28,GLY-30,GLU-31,TYR-32,ILE-33,PRO-34,THR-35
Geldanamycin	HSP90AB1	GLE-47,LEU-48,ASN-51SER-52,ASP-54,ALA-55,LEU-56,LYS-58,ILE-96,GLY-97,MET-98,THR-98,ASP-102,ASN-106,PHE-134,GLY-135,VAL-136,GLY-137,HIS-154
Eugenol	ESR1	MET-342,LEY-345,LEU346,THR-347,ASN-348,ALA-350,ILE-386,LEU-387,GLY-390,VAL-392,ARG-394,PHE-404,MET-421,ILE-424,MET-517,LEU-525

**Table 4:** Binding free energy of ligand-receptor complexes and their corresponding interaction energies

**Drug**	**Receptor**	**AutoDock-4** **(Kcal/mole)**	**RMSD in Å**	**Hydrogen bonding interactions**		
				**Interacting Residues**	**Distance (Å)**	**Angle (Degree)**
Lapatinib	ERBB2	-10.59	1.16	LEU-785	3.160	87.979
				MET-801	3.149	113.787
Azathioprine	RAC1	-9.32	2.0	GLY-15	2.991	105.258
				THR-17	3.172	95.738
Geldanamycin	HSP90AB1	-8.83	1.93	GLY-137	3.105	-
				PHE-138	3.065	-
Eugenol	ESR1	-5.23	1.21	GLU-353	1.791	148.321
				ARG-394	2.885	-

## Conclusion

This study showed that the investigation of interactions between targets with drugs at the system-level as well as in the context of biological and disease networks could resulted in drugs and targets prioritization. By integrative systems biology approach, we identified thirteen drugs which targeted eight central proteins in PBMCs and pancreatic β-cells QQPPI networks and represented significant associations with diabetes. One drug (imatinib) is being explored in clinical trial for T1D, which shown the robustness of our strategy. Moreover, we prioritized drug-targets such as melatonin, resveratrol, eugenol, lapatinib, geldanamycin and azathioprine which interacted with ESR1, ERBB2, HSP90AB1 and RAC1, respectively and shown associations in diabetes treatment in experimental models of T1D. Lastly, the interaction of some drug-targets was predicted by molecular docking analysis. After further validation, these prioritized targets and drugs could be a potential candidate for the targeted therapy and facilitate the drug discovery for patients with T1D. 
